# Borderline personality disorder vs. schizophrenia spectrum disorders in young people recruited within an “Early Intervention in Psychosis” service: clinical and outcome comparisons

**DOI:** 10.1007/s00406-024-01772-5

**Published:** 2024-03-12

**Authors:** Lorenzo Pelizza, Emanuela Leuci, Emanuela Quattrone, Silvia Azzali, Giuseppina Paulillo, Simona Pupo, Pietro Pellegrini, Lorenzo Gammino, Arianna Biancalani, Marco Menchetti

**Affiliations:** 1 Department of Biomedical and Neuromotor Sciences, Psychiatry Institute, “Alma Mater Studiorum” Università degli Studi di Bologna, Via Pepoli 5, 40123 Bologna, BO Italy; 2 Department of Mental Health and Pathological Addictions, Azienda USL di Parma, Largo Palli 1/a, 43100 Parma, Italy; 3 Department of Mental Health and Pathological Addictions, Azienda USL-IRCCS di Reggio Emilia, Via Amendola 2, 43100 Reggio Emilia, Italy; 4 Division of Pain Medicine, Department of Medicine and Surgery, Azienda Ospedaliero-Universitaria di Parma, Via Gramsci 14, 43100 Parma, Italy; 5 Department of Mental Health and Pathological Addictions, Azienda USL di Bologna, Via Castiglione 29, 40124 Bologna, Italy

**Keywords:** Borderline personality disorder, Early psychosis, Schizophrenia spectrum disorder, Early intervention

## Abstract

Borderline Personality Disorder (BPD) is under-recognized in First-Episode Psychosis (FEP) and its psychotic manifestations are difficult to differentiate from Schizophrenia Spectrum Disorders (SSD). The aim of this investigation was to compare clinical, sociodemographic, and outcome characteristics between FEP patients with BPD vs. FEP subjects with SSD both at baseline and across a 2-year follow-up period. Participants completed the Health of the Nation Outcome Scale (HoNOS), the Positive And Negative Syndrome Scale (PANSS), and the Global Assessment of Functioning (GAF) scale both at entry and every 12 months during the follow-up. A mixed-design ANOVA model was conducted to investigate the temporal stability of clinical scores within and between the two subgroups. Among 356 FEP participants, 49 had a BPD diagnosis. Compared to FEP/SSD (*n* = 307), FEP/BPD patients showed higher prevalence of employment, current substance use, and past attempted suicide. They had a lower equivalent dose of antipsychotic medication at entry and lower levels of negative symptoms. Finally, they had a higher 2-year drop-out rate and a significant improvement in psychopathological scores limited to the first year of treatment. BPD as categorical entity represents a FEP subgroup with specific clinical challenges. Appropriate treatment guidelines for this FEP subgroup are thus needed.

**Supplementary Information:**

The online version contains supplementary material available at 10.1007/s00406-024-01772-5.

## Introduction

Historically, the term “borderline” was used to describe patients specifically experiencing both neurotic and psychotic symptoms [1], who were often resistant to psychoanalytic approach [2]. However, it was only with the IV edition of the Diagnostic and Statistical Manual of mental disorders (DSM-IV) [3] that psychotic features were listed among the diagnostic criteria for Borderline Personality Disorder (BPD), albeit in the form of stress-related, transient paranoid ideation and/or dissociative symptoms (i.e., depersonalization, derealization) [4]. From then on, the DSM categorical criteria for BPD remained substantially unchanged [5]. More recently, the alternative dimensional DSM-5 model of personality disorders did not consider psychotic symptoms as central psychopathological characteristics of BPD, but “psychoticism” traits could be added as specifier to further clarify the features of the disorder [6].

### Psychotic symptoms in BPD

Psychotic symptoms in BPD are relatively common and are often distressing and challenging to treat [7]. Their clinical presentation is more heterogeneous than what current classification systems suggest [8] and is not limited to the long-standing notion that they are transient, stress-dependent, and confined to dissociative symptoms and paranoia.

In this respect, auditory verbal hallucinations (AVHs) are the most common psychotic manifestations in BPD [9, 10], with reported prevalence rates of 25–50% [11–13]. Moreover, they are often very similar to those experienced in people with Schizophrenia Spectrum Disorders (SSD), especially in terms of location, duration, and frequency [14].

Albeit attracting less research attention, delusions in BPD have reported prevalence rates of 15–30% [15] and frequently involve paranoid contents [16] that cannot be easily differentiated from delusions manifesting in SSD [17]: indeed, they are often disconnected from shared reality and unrelated to specific stressful events [18], sometimes persisting in the absence of ongoing stressors [19].

Psychotic symptoms in BPD are currently considered as psychopathological indicators of illness severity and poor prognosis [20]. Thus, it is crucial to detect them as soon as possible, especially at BPD clinical onset, so as to prevent the development of a severe mental illness in the future (including SSD). In this respect, it was reported that psychotic symptoms in BPD are associated with a higher number of BPD criteria, high levels of psychopathology, more co-occurring mental disorders (such as substance use disorder, mood disorders, and post-traumatic stress disorder), high rates of new hospitalization and suicidal behavior, and quicker re-admissions to acute psychiatric inpatient care after discharge [21].

As adolescence and young adulthood are relevant sensitive life periods in which both BPD psychopathology and psychotic symptoms usually emerge for the first time [22], clinical interest on BPD has recently focused on patients with First-Episode Psychosis (FEP). However, evidence on BPD prevalence and its associations with psychotic features in young FEP people is still poor [23], especially because at the early phases of psychosis it is difficult to differentiate whether psychotic symptoms are inherent to BPD or to a primary psychotic disorder [24]. Thus, this remains a research topic urgently requiring more attention, especially because psychotic features in BPD could require appropriate treatment guidelines outside those specifically developed for FEP.

Starting from this background, the aims of this retrospective cohort study were as follows:

- To compare baseline clinical, sociodemographic, and treatment characteristics between FEP patients with BPD and those with SSD, all recruited and treated within an “Early Intervention in Psychosis” (EIP) service.
- To compare the longitudinal course of clinical and outcome parameters (i.e., new hospitalization, drop-out condition, new suicide attempt/self-harm behavior) between the two FEP subgroups along a 2-year follow-up period.

To the best of our knowledge, no investigation specifically comparing BPD and SSD in FEP subjects has been published in the literature to date.

## Methods

### Setting and subjects

FEP participants were enrolled within the “Parma Early Psychosis” (Pr-EP) program between January 2013 and December 2021. The Pr-EP is a specialized, diffuse EIP protocol specifically implemented in all adult and adolescent mental healthcare services of the Parma Department of Mental Health [25].

Inclusion criteria of this research were as follows: (a) specialist help-seeking request; (b) age 18–35 years; (c) FEP patients enrolled in the Pr-EP program; (d) presence of BPD or SSD as final primary diagnosis in accordance with DSM-IV-TR diagnostic criteria [26]; and (e) a “Duration of Untreated Psychosis” (DUP) of < 2 years. This DUP was specifically selected as inclusion criterion in this investigation to comply with the usual time range limit considered before the access to care within the EIP research paradigm [27, 28], as well as to include FEP subjects early in their psychopathological trajectory.

Exclusion criteria were as follows: (a) past full-blown psychotic episode within DSM-IV-TR diagnosis of both affective and non-affective psychosis; (b) past exposure to antipsychotic drug or current antipsychotic intake for more than 2 months prior to the Pr-EP recruitment; (c) neurological disease or any other medical condition presenting with psychiatric symptoms; and (d) known intellectual disability (i.e., intelligence quotient < 70). Specifically, we considered past exposure to antipsychotic medication (i.e., at any time and dosage prior to the Pr-EP enrollment) as “functional equivalent” of past psychotic episode. This was in accordance with the psychosis threshold as originally defined by Yung and co-workers [29] within the EIP paradigm (i.e., “essentially that at which an antipsychotic treatment would probably be started in the common clinical practice”). Finally, a current antipsychotic intake for less than 2 months was selected because it is the time range specifically defined to complete the assessment phase in the Pr-EP protocol.

All individuals and their parents (if minors) gave their written informed consent prior to their inclusion in the study. Local ethical approvals were obtained for the research (AVEN protocol n. 36,102/09.09.2019). This investigation was conducted in accordance with the ethical standards of the 1964 Declaration of Helsinki and its later amendments.

### Instruments and measures

The clinical assessment of this investigation included the Positive and Negative Syndrome Scale (PANSS) [30], the Global Assessment of Functioning (GAF) scale [26], and the Health of the Nation Outcome Scale [31]. These instruments were completed at baseline and every 12 months during the follow-up period by trained Pr-EP team members. Regular supervision sessions and scoring workshops ensured their inter-rater reliability [32].

The PANSS is a clinical interview commonly used to assess psychopathology, also in early psychosis [33–35]. As proposed by Shafer and Dazzi [36], we considered five main psychopathological factors: “Negative Symptoms,” “Affect” (“Depression/Anxiety”), “Positive Symptoms,” “Disorganization,” and “Resistance/Excitement.”

The GAF is frequently used to evaluate daily functioning in individuals with psychosis, including young FEP patients [37, 38].

The HoNOS was specifically developed to assess clinical and social outcomes in people with severe mental illness, including young populations with early psychosis [38, 39]. As proposed by Gale and Boland [40], we considered four main outcome domains: “Psychiatric Symptoms,” “Impairment,” “Social Problems,” and “Behavioral Problems.”

A sociodemographic and clinical chart (collecting information on gender, years of education, age at entry, ethnic group, housing/marital and employment status, DUP, source of referral, past hospitalization, previous specialist contact, past self-harm/suicidal behavior, and current substance use) was also completed at baseline [41]. Specifically, the DUP was defined as the time interval (in months) between the onset of psychotic symptoms and the initiation of the first antipsychotic treatment [42]. The presence of frank psychotic symptoms was defined according to the “Comprehensive Assessment of At-Risk Mental States” (CAARMS) psychosis threshold criteria [29]. Data on DUP and first pharmacological treatment were collected through clinical interviews with patients and/or family members, and/or consulting their medical records. The term “suicide attempt” was used to define a potentially injurious, self-inflicted behavior without fatal outcome, for which there was an implicit or explicit intent to die [43]. It was differentiated from undetermined acts of deliberate self-harm or intoxication with alcohol or drugs without evidence of intent to die (referred to as “self-harm” behaviors) [44].

### Procedures

The presence of FEP was formulated in accordance with CAARMS (“Comprehensive Assessment of At-Risk Mental States”) criteria [29]. Moreover, the DSM-IV-TR primary diagnoses were formulated both at baseline (“initial” diagnosis) and at the end of the follow-up (“final” diagnosis). As for participants who did not complete the follow-up period, final diagnoses were defined together with clinicians treating and managing FEP patients (see Supplementary Materials (Tables S1, S2) for details). Specifically, primary diagnoses were defined by at least two trained Pr-EP team members on each occasion in accordance with the DSM-IV-TR diagnostic criteria, using both the Structured Clinical Interview for DSM-IV-TR axis I Disorders (SCID-I) [45] and the Structured Clinical Interview for DSM-IV axis II personality disorders (SCID-II) [46]. Participants with BPD as final diagnosis were then included in the FEP/BPD subgroup. Patients with SSD (i.e., schizophrenia, schizoaffective disorder, and schizotypal personality disorder) as final diagnosis were included in the FEP/SSD subgroup.

According to symptom severity, FEP subjects were provided with a 2-year comprehensive intervention protocol including psychopharmacological therapy and a multicomponent psychosocial treatment (combining psychoeducational sessions for family members, intensive recovery-oriented case management, and individual psychotherapy inspired on cognitive-behavioral principles), as suggested by current EIP guidelines on the topic [47–49]. Low-dose atypical antipsychotic drug was used as first-line pharmacological therapy [50]. Mood stabilizers, serotonin selective reuptake inhibitors, and/or benzodiazepines could also be used to treat mood changes, anxiety, and insomnia [51].

As for between-group comparisons, clinical characteristics were examined both at baseline and every 12 months along the follow-up period, together with sociodemographic features and the acceptance of Pr-EP treatment proposals at entry. We also compared the two subgroups on four main outcome indicators across the follow-up period (i.e., drop-out rate, new hospital admission, new suicide attempt and new self-harm behavior).

### Statistical analysis

Data were analyzed using the Statistical Package for Social Science (SPSS) for Windows, version 15.0 [52]. All tests were two-tailed with a significance level set at 0.05. In inter-group comparisons, continuous parameters were examined using the Mann–Whitney *U* test and categorical measures using the Chi-square test. Kaplan–Meier survival analysis to longitudinally compare outcome indicators between the two subgroups was also performed. This ensured to consider the different time duration of individual follow-ups and participants who dropped out before the end of follow-up [53]. Finally, a mixed-design ANOVA model (with post hoc Bonferroni correction for multiple comparisons) was performed to evaluate the temporal stability of PANSS, GAF, and HoNOS scores within and between the two subgroups along the follow-up period [54].

## Results

Forty-nine (13.3%) out of 356 FEP participants had BPD as final diagnosis and were included in the FEP/BPD subgroup. The remaining 307 individuals were included in the FEP/SSD subgroup: they were affected by schizophrenia (*n* = 249 [81.1%]), schizoaffective disorder (*n* = 29), and schizotypal personality disorder (*n* = 29) (see Supplementary Materials (Table S3) for baseline diagnosis). Sociodemographic and clinical features of the two subgroups are shown in Table 1.

**Table 1:** Sociodemographic and clinical characteristics of the two FEP subgroups (*n* = 356)

Variable	BPD	SSD	*χ*^2^*/z*	*p*
	(*n* = 49)	(*n* = 307)		
Gender (male)	33 (66.7%)	205 (66.8%)	0.006	0.937
Age at entry (in years)	24.39 ± 6.43	25.43 ± 6.18	− 1.149	0.251
Education (in years)	11.96 ± 2.98	11.27 ± 2.75	− 1.39	0.164
Ethnic group (white Caucasian)	42 (85.7%)	256 (83.4%)	0.168	0.682
Migrant status	11 (22.4%)	75 (24.4%)	0.091	0.764
Civil status				
Single	45 (91.8%)	282 (91.9%)	0.001	0.996
Married/partnership	3 (6.1%)	19 (6.2%)	0.001	0.986
Separated/divorced	1 (2.0%)	6 (2.0%)	0.002	0.999
Living status				
Alone	5 (10.2%)	22 (7.2%)	0.556	0.396
Living with partners	8 (16.3%)	53 (17.3%)	0.026	0.872
Living with parents	35 (71.4%)	219 (71.3%)	0.001	0.989
Living in residential facility	1 (2.0%)	11 (3.6%)	0.309	0.999
Homeless	0 (0.0%)	2 (0.7%)	0.001	0.998
Occupation				
Unemployed	21 (42.9%)	171 (55.7%)	2.805	0.098
Employed	17 (37.4%)	60 (19.5%)	5.721	**0.017**
Student	11 (22.4%)	76 (24.8%)	0.122	0.727
DUP (in months)	6.15 ± 6.44	10.48 ± 10.69	− 2.608	**0.009**
Source of referral				
Primary care	14 (28.6%)	102 (33.2%)	0.417	0.519
Other mental health services	13 (26.5%)	43 (14.0%)	5.000	**0.025**
Emergency room	10 (20.4%)	90 (29.3%)	1.660	0.198
Family members	6 (12.2%)	36 (11.7%)	0.011	0.917
Self-referral	5 (10.2%)	25 (8.1%)	0.233	0.584
School/social services	1 (2.0%)	11 (14.0%)	0.309	0.999
Past hospitalization	14 (28.6%)	137 (44.6%)	4.459	**0.035**
Past specialist contact	21 (42.9%)	133 (43.3%)	0.004	0.951
Age at first past specialist contact	17.80 ± 6.69	20.89 ± 7.09	− 1.897	0.058
Substance misuse (at entry)	28 (57.1%)	107 (34.9%)	8.918	**0.003**
Past suicide attempt	8 (16.3%)	23 (7.5%)	4.149	**0.042**
Past self-harm	20 (40.8%)	110 (35.8%)	0.453	0.501

Frequencies (percentages), mean ± standard deviation, Chi-squared test (*χ*^2^), and Mann–Whitney *U* test (*z*) values are reported. Statistically significant *p* values are in bold

*BPD* Borderline personality disorder; *SSD* schizophrenia spectrum disorders; *DUP* duration of untreated psychosis

### Baseline comparisons

Compared to FEP/SSD, FEP/BPD patients showed a shorter DUP, higher rates of employment, current substance use, past suicide attempt, and other general mental healthcare services as primary sources of referral (i.e., psychological counseling services for adolescents or young adults, pathological addiction services, private psychiatrists, and psychologists), as well as a lower rate of previous hospitalization (Table 1). They also had a lower PANSS “Negative Symptoms” factor score and a lower baseline prescription rate of antipsychotic medication (Table 2).

**Table 2:** Psychopathological and Pr-EP treatment characteristics of the two FEP subgroups (*n* = 356)

Variable	BPD (*n* = 49)	SSD (*n* = 307)	*Χ*^2^*/z*	*p*
Baseline PANSS “positive” factor	18.00 ± 4.66	16.57 ± 6.15	− 1.466	0.143
Baseline PANSS “negative” factor	21.19 ± 7.98	26.03 ± 9.10	− 2.708	**0.007**
Baseline PANSS “disorganization” factor	19.12 ± 8.32	21.08 ± 7.85	− 1.481	0.139
Baseline PANSS “affect” factor	17.96 ± 5.05	16.03 ± 5.58	− 1.596	0.111
Baseline PANSS “resistance” factor	8.27 ± 3.48	9.42 ± 4.66	− 0.913	0.361
Baseline PANSS total score	87.96 ± 19.61	92.39 ± 24.14	− 1.288	0.198
Baseline GAF score	44.72 ± 12.33	44.29 ± 9.72	− 0.107	0.915
Baseline HoNOS “behavioral problems” domain	4.10 ± 2.50	3.61 ± 2.41	− 1.411	0.158
Baseline HoNOS “impairment” domain	2.82 ± 11.90	3.20 ± 2.04	− 1.171	0.241
Baseline HoNOS “psychiatric symptoms” domain	10.02 ± 3.73	9.98 ± 3.46	− 0.099	0.921
Baseline HoNOS “social problems” domain	7.10 ± 3.62	7.95 ± 3.87	− 1.435	0.151
Baseline HoNOS total score	24.04 ± 7.99	24.74 ± 8.67	− 0.624	0.532
Baseline antipsychotic prescription	38 (77.6%)	271 (88.3%)	4.240	**0.039**
Baseline Antidepressant prescription	6 (12.2%)	37 (12.1%)	0.001	0.969
Baseline mood stabilizer prescription	4 (8.2%)	20 (6.5%)	0.183	0.669
Baseline benzodiazepine prescription	17 (34.7%)	101 (32.9%)	0.061	0.804
Baseline equivalent dose of risperidone (mg/day)	2.35 ± 2.56	3.16 ± 2.77	− 2.728	**0.006**
Baseline individual psychotherapy acceptance	41 (83.7%)	249 (81.1%)	0.184	0.668
Baseline family psychoeducation acceptance	33 (67.3%)	206 (67.1%)	0.001	0.973
Baseline case management acceptance	38 (77.6%)	254 (82.7%)	0.770	0.380

Frequencies (percentages), mean ± standard deviation, Chi-squared test (*χ*^2^) (and adjusted residuals), and Mann–Whitney *U* test (*z*) values are reported. Statistically significant *p* values are in bold. Holm–Bonferroni corrected *p* values are reported

*BPD* Borderline personality disorder; *SSD* schizophrenia spectrum disorder; *Pr-EP* parma early psychosis program; *PANSS* positive and negative syndrome scale; *GAF* global assessment of functioning; *HoNOS* health of the nation outcome scale.

### Longitudinal comparisons

Two hundred and forty-six participants (69.2% of the FEP total sample) completed the follow-up (see Supplementary Materials (Table S1) for details). Forty-five out of 110 FEP patients not completing the follow-up dropped out the Pr-EP program (18 of them during the first 12 months of observation) and 65 individuals were disengaged in accordance with the treatment staff (34 for clinical improvement and 31 because moving outside the catchment area and they could not be contacted for the follow-up assessments). Specifically, such 65 FEP subjects were not considered as dropped out.

**Fig. 1:**
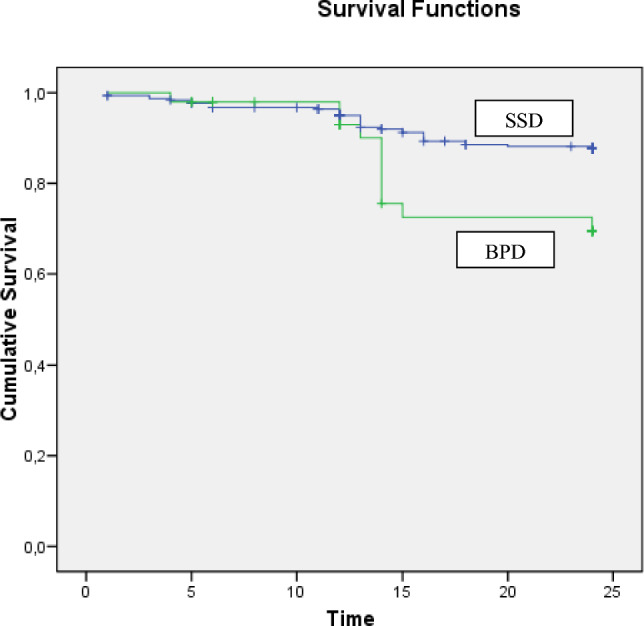
Kaplan–Meier survival analysis results: comparison of 2-year drop-out rate between the two FEP subgroups (*n* = 356). *BPD *Borderline personality disorder; *SSD *schizophrenia spectrum disorder

Kaplan–Meier survival analysis results showed higher 2-year drop-out rate and lower mean survival estimate in FEP/BPD compared to FEP/SSD participants (20.946 [Standard Error = 0.880] vs 23.657 [0.262] in months; *χ*^2^ = 7.566; *p* = 0.006) (see Fig. 1 and Supplementary Materials (Table S4) for details). No statistically significant differences in terms of 2-year new hospital admission, new attempted suicide, and new self-harm behavior rates were observed (see Supplementary Materials (Tables S5–S7) for details).

Mixed-design ANOVA results on repeated parameters (i.e., “within-subject” effects) showed a significant effect of time on all PANSS, HoNOS, and GAF scores (Table 3). However, after 12 months of follow-up, the two FEP subgroups had a similar statistically significant decrease in all PANSS and HoNOS dimension scores, except for PANSS “Negative Symptoms” and “Affect” factor subscores that showed a lower statistical significance in FEP/BPD compared to FEP/SSD participants (Table 4). Moreover, during the first year of intervention, FEP/BPD patients had no statistically significant improvement in PANSS “Resistance” factor subscore and a lower statistically relevant increase in GAF score compared to FEP/SSD individuals.

**Table 3:** Mixed-design ANOVA results: psychopathological and outcome characteristics across the 2-year follow-up period in patients with BDP versus patients with SSD

Variable	Time effect				Group effect (BPD vs. SSD)				Interaction effect (time x group)			
	df	*F*	*p*	*η*^2^	df	*F*	*p*	*η*^2^	df	*F*	*p*	*η*^2^
PANSS positive factor	1.6	23.013	**0.0001**	0.143	1	0.199	0.656	0.001	1.6	1.985	0.148	0.014
PANSS negative factor	1.9	19.166	**0.0001**	0.122	1	1.561	0.214	0.011	1.9	0.539	0.577	0.004
PANSS disorganization factor	1.5	34.125	**0.0001**	0.199	1	0.015	0.904	0.001	1.5	0.413	0.606	0.003
PANSS affect factor	1.5	30.618	**0.0001**	0.182	1	0.742	0.391	0.005	1.5	0.340	0.661	0.002
PANSS resistance factor	2	6.424	**0.003**	0.044	1	0.03	0.862	0.001	2	4.378	**0.013**	0.031
PANSS total score	1.7	40.165	**0.0001**	0.227	1	0.061	0.689	0.001	1.7	1.066	0.338	0.008
HoNOS “behavioral problems” domain	1.6	55.021	**0.0001**	0.182	1	1.988	0.160	0.008	1.6	0.066	0.902	0.001
HoNOS “impairment” domain	1.5	45.806	**0.0001**	0.156	1	0.454	0.501	0.002	1.5	1.696	0.191	0.007
HoNOS “psychiatric symptoms” domain	1.7	89.143	**0.0001**	0.264	1	2.200	0.059	0.017	1.7	1.249	0.285	0.005
HoNOS “social problems” domain	1.6	49.755	**0.0001**	0.167	1	0.479	0.489	0.002	1.6	0.148	0.820	0.001
honos total score	1.5	112.883	**0.0001**	0.314	1	0.834	0.362	0.003	1.5	0.684	0.472	0.003
GAF	1.8	62.055	**0.0001**	0.293	1	0.033	0.856	0.001	1.8	0.159	0.834	0.001

As all Mauchly’s tests of sphericity are statistically significant (*p* < 0.05), Greenhouse–Geisser corrected degrees of freedom to assess the significance of the corresponding *F* value are used. Statistically significant *p* values are in bold

*ANOVA* Analysis of variance; *BPD* borderline personality disorder; *SSD* schizophrenia spectrum disorders; *PANSS* positive and negative syndrome scale; *df* degrees of freedom; *F* F statistic value; *GAF* global assessment of functioning; *HoNOS* health of the nation outcome scale; *p* statistical significance; *η*^2^ partial eta squared

**Table 4:** Post hoc test on psychopathological and outcome characteristics across the 2-year follow-up period in patients with BPD and SSD

Variables in BDP patients	T0 vs. T1					T1 vs. T2				
	MD	SE	*p*	95% CI	*d*	MD	SE	*p*	95% CI	*d*
PANSS positive factor	7.44	1.48	**0.0001**	4.32, 10.57	0.99	– 1.18	0.87	0.335	– 3.78, 1.42	–
PANSS negative factor	6.27	1.77	**0.002**	2.54, 10.01	0.11	– 0.09	0.61	0.973	– 5.87, 5.69	–
PANSS disorganization factor	5.89	1.34	**0.0001**	3.05, 8.73	0.99	1.18	0.49	0.403	– 1.83, 4.20	–
PANSS affect factor	5.17	1.34	**0.001**	2.34, 7.99	0.91	0.09	0.93	0.952	– 3.22, 3.40	–
PANSS resistance factor	1.22	0.69	0.096	0.024, 2.68	–	– 0.64	0.44	0.554	– 2.93, 1.68	–
PANSS total score	27.00	5.36	**0.0001**	15.70, 38.30	0.99	– 1.09	0.09	0.884	– 17.28, 15.09	–
HoNOS “behavioral problems”	2.15	0.43	**0.0001**	1.27, 3.30	0.79	0.64	0.44	**0.036**	0.05, 1.23	0.44
HoNOS “impairment”	1.61	0.28	**0.0001**	1.06, 2.17	0.93	– 0.08	0.15	0.731	– 0.55, 0.39	–
HoNOS “psychiatric symptoms”	4.72	0.62	**0.0001**	3.45, 5.98	0.99	0.80	0.11	0.211	– 0.48, 2.08	–
HoNOS “social problems”	2.82	0.53	**0.0001**	1.76, 3.88	0.86	0.64	0.89	0.103	– 0.14, 1.42	–
HoNOS total score	11.31	1.45	**0.0001**	8.36, 14.25	0.991	2.00	0.68	0.091	– 0.34, 4.34	–
GAF	– 15.70	3.93	**0.001**	– 23.92, – 7.47	0.89	– 4.61	0.65	0.246	– 12.87, 3.63	–

Variables in SSD patients	T0 vs. T1					T1 vs. T2				
	MD	SE	*p*	95% CI	*d*	MD	SE	*p*	95% CI	*d*
PANSS positive factor	4.09	0.43	**0.0001**	3.12, 5.07	0.64	1.56	0.35	**0.0001**	0.87, 2.25	0.39
PANSS negative factor	5.57	0.02	**0.0001**	4.19, 6.95	0.62	2.74	0.69	**0.0001**	1.37, 4.10	0.35
PANSS disorganization factor	4.84	0.74	**0.0001**	3.96, 4.93	0.84	1.41	0.36	**0.0001**	0.69, 2.14	0.34
PANSS affect factor	4.22	0.66	**0.0001**	3.51, 4.93	0.91	1.19	0.27	**0.0001**	0.66, 1.73	0.39
PANSS resistance factor	2.02	0.70	**0.0001**	1.46, 2.58	0.55	1.09	0.21	**0.0001**	0.68, 1.51	0.46
PANSS total score	21.70	1.37	**0.0001**	18.12, 25.28	0.93	8.63	1.46	**0.0001**	5.74, 11.52	0.52
HoNOS “behavioral problems”	1.55	0.12	**0.0001**	1.31, 1.80	0.77	0.77	0.12	**0.0001**	0.54, 1.00	0.44
HoNOS “impairment”	1.02	0.61	**0.0001**	0.83, 1.21	0.63	0.49	0.08	**0.0001**	0.33, 0.64	0.41
HoNOS “psychiatric symptoms”	3.03	0.67	**0.0001**	2.60, 3.46	0.76	1.66	0.17	**0.0001**	1.33, 1.99	0.66
HoNOS “social problems”	2.43	0.19	**0.0001**	2.06, 2.81	0.76	0.77	0.16	**0.0001**	0.46, 1.08	0.32
HoNOS total score	8.00	0.73	**0.0001**	7.09, 8.91	0.99	3.69	0.36	**0.0001**	2.98, 4.40	0.68
GAF	– 12.16	1.20	**0.0001**	– 13.95, – 10.36	0.99	– 4.61	0.73	**0.0001**	– 6.05, – 3.17	0.54

Statistically significance *p* values are in bold. Bonferroni corrected *p* values are reported

*BDP* Borderline personality disorder; *SSD* schizophrenia spectrum disorder; *PANSS* positive and negative syndrome scale; *HoNOS* health of the nation outcome scale; *GAF* global assessment of functioning; *MD* mean difference; *SE* standard error; *T0* baseline assessment time; *T1* 1-year assessment time; *T2* 2-year assessment time; *p* statistical significance; *95% CI* 95% Confidence Intervals; *d* Cohen’s *d* for size effect

During the second year of follow-up, compared to FEP/SSD, FEP/BPD participants had no statistically significant improvement in all PANSS, GAF, and HoNOS scores, except for the HoNOS “Behavioral Problems” domain subscore that showed a lower statistically relevant decrease in the FEP/BPD subgroup (Table 4).

Mixed-design ANOVA results on “between-subject” effects showed no statistically significant group effect. However, a statistically relevant “time x group” interaction effect was found in the PANSS “Resistance” dimension score (Table 4), which showed a significant longitudinal improvement exclusively in the FEP/SSD subgroup.

## Discussion

Compared to FEP/SSD, FEP/BPD patients showed a higher employment rate at entry, suggesting a better baseline occupational functioning. This is in line with the traditional descriptions of “borderline states,” such as subjects with “ambulatory schizophrenia” [55] or “pseudoneurotic schizophrenia” [56], who «…appear normal in all respects, go to business, and may have a position and keep it» [57]. Differently, other authors found that BPD individuals with psychotic symptoms showed a relevant social functioning decline and did not significantly differ from patients with schizophrenia [58]. Our reported better functioning result may be related to a patient recruitment in an early illness stage.

Compared to FEP/SSD, our FEP/BPD participants showed higher baseline rates of current substance use and past suicide attempt. These findings are in line with what was found in the “Early Psychosis Prevention and Intervention Centre” (EPPIC) cohort, suggesting that FEP patients with borderline psychopathology were more likely to have self-harm problems and substance use disorder at presentation than individuals with FEP alone [59]. Therefore, these problematic behaviors seem to more frequently characterize the onset of FEP with comorbid BPD rather than first-episode SSD. However, the question remains how much psychoactive drugs influence the onset and course of psychotic manifestations in BPD [60].

Our FEP/BPD participants had a higher baseline rate of “other mental healthcare services” as primary source of Pr-EP referral, together with a shorter DUP at entry. These results suggest that the onset of a psychological distress in BPD subjects more often induces them to contact general mental healthcare centers (such as pathological addiction teams, psychological counselors for young people and adolescents, private psychologists, and psychiatrists) rather than specialized EIP centers. Only the intensification of psychotic features probably then induces a prompt referral to EIP programs, also shortening the DUP. In this sense, BPD psychopathology may paradoxically represent a positive factor for a timely referral to EIP services and for an early intervention on psychotic symptoms (especially when compared to FEP/SSD patients). This is crucial for improving prognosis and outcomes in FEP patients, especially in adolescence [61]. The DUP reduction could also be related to their higher social ability and their more effective help-seeking behavior, as documented by lower baseline levels of negative symptoms [62] and a lower rate of previous hospitalization at entry. This latter finding is in line with what was observed in the EPPIC cohort [59].

As for psychopathological characteristics, lower baseline levels in negative symptoms specifically characterized our FEP/BPD participants, differentiating them from those with first-episode SSD. As proposed by Bleuler [63], negative symptoms are central SSD clinical features already at the illness onset. Indeed, our FEP subgroups showed comparable baseline levels of positive symptoms and disorganization, although FEP/BPD patients had a lower prescription rate of antipsychotic medication at baseline. This pharmacological result may be due to low motivation in taking pharmacological therapy or low adherence to treatment in FEP/BPD patients [64], as well as to a lower propensity of clinicians to pharmacologically treat FEP/BPD patients considering the presumed clinical transience of psychotic symptoms, a baseline functioning not grossly deteriorated, and the peremptory psychopharmacological recommendations of the current BPD guidelines [65]. Moreover, their higher baseline rate of substance abuse may lead the clinicians to think that psychotic features are dealing with secondary phenomena mainly related to drug abuse, and to consider them more “benign” despite the presence of primary psychotic symptoms.

On a strictly psychopathological point of view, our FEP/BPD participants did not exclusively show paranoid ideation and dissociative symptoms (as defined in the DSM-5 9th diagnostic criteria for BPD) [5]: indeed, 51% of them showed hallucinatory behaviors and 88% different (not only persecutory) delusional themes. These findings are in line with previous evidence in BPD individuals [13] and have important clinical implications. As psychotic features in BPD are not limited to dissociative symptoms and paranoia, the BPD criteria necessarily require a critical revision [66]. The historical “tale” that psychotic manifestations in BPD are somehow transient and not real is a “disrespectful myth” that is inconsistent with subjective experiences of BPD individuals [67]. Moreover, it is a “false dichotomy” to consider that these individuals may have either BPD or psychosis, because they may have both. In this respect, some studies comparing clinical populations of BPD and SDD subjects reported that a significant part of their samples (ranging between 15 and 20%) had the disorders occurring [68–71].

As in bipolar disorder or major depression, psychotic features may be markers of illness severity [72] and identify a specific subgroup of BPD patients [73, 74]. However, the question remains whether considering psychotic symptoms in BPD as simple indicators of clinical severity and poor outcome, or as “core” features of a specific BPD subgroup to place within the psychosis spectrum disorder rather than within cluster B personality disorders. In this respect, future studies examining BPD psychopathology, basic symptoms, and anomalous self-experiences in young people at clinical high risk of psychosis could clarify these psychopathological hypotheses.

As for outcome indicators, our FEP/BPD patients showed a higher 2-year drop-out rate compared to FEP/SSD participants. Comorbid BPD diagnosis may thus be considered as a negative prognostic factor in terms of retention in care of FEP patients within EIP programs. So, it is thus crucial to implement treatment strategies to decrease their service disengagement. Specifically, it could be helpful to routinely perform an in-depth diagnostic assessment for early identifying BPD psychopathology in FEP populations, both at the enrollment within EIP protocols and during the follow-up. Moreover, it could be useful to strengthen and maintain treatment motivation in FEP/BPD subjects, also through increasing focused psychoeducational sessions. Finally, it is of clinical relevance that individual therapeutic-rehabilitation programs for FEP/BPD patients define specific interventions (such as the “Good Psychiatric Management”) [75], as well as realistic short-term treatment goals (more easily and quickly achievable within few months). Indeed, this higher drop-out risk in our FEP/BPD individuals happened even in the presence of no between-group difference in terms of baseline acceptance of psychosocial proposals and may be associated with specific BPD psychopathological features (such as mood fluctuations, instability in therapeutic alliance, and relationships) and/or the lack of clinical improvement over time (especially during the second year of treatment). As an alternative, BPD participants may have dropped out the Pr-EP program on their own intention due to improvement in their symptoms. In the EPPIC cohort, FEP individuals with BPD psychopathology differently showed poorer access to treatment than the FEP alone subgroup [76]. In this respect, the easier access in Italy to generalist mental healthcare services may be considered a facilitating factor for treatment compared to a model based on specialized stand-alone programs.

Our mixed-design ANOVA results showed a significant effect of time on all functioning, psychopathological and outcome parameters in both subgroups. However, after one year of treatment, FEP/BPD participants had a lower statistical significance in improvements on daily functioning and negative symptoms than FEP/SSD subjects. Moreover, during the second year, they notably showed no statistically relevant effect on functioning, psychopathological and outcome variables, except for the HoNOS “Behavioral Problems” domain subscore. These findings suggest less intensive beneficial effects of EIP interventions in FEP/BPD than in FEP/SSD patients. As EIP interventions were developed on the treatment of schizophrenia, it is necessary to differentiate EIP protocols according to different diagnostic categories, and to develop more adapted care for FEP/BPD patients. A pilot study on 16 young patients with FEP and BPD showed the feasibility and efficacy of a hybrid psychosocial program combining elements of early intervention for BPD within a specialized FEP intervention [76, 77].

Compared to FEP/SSD, FEP/BPD participants showed no significant longitudinal improvement in PANSS “Resistance/Excitement” factor score (including hostility and uncooperativeness features). This supports a lower beneficial effect of traditional EIP interventions on treatment adherence of FEP/BPD patients, potentially increasing their drop-out rate [78]. Specific strategies for strengthening and maintaining treatment motivation in FEP/BPD subjects are thus needed.

### Limitations

A first limitation of our research was the relatively small sample size (*n* = 49) of the FEP/BPD subgroup. Future studies on larger FEP populations also meeting BPD criteria are thus needed. Moreover, BPD patients treated in an EIP program were unlikely to be representative of all or even most individuals with BPD. As they more probably represented a selected BPD subgroup with relatively higher levels of psychosis, this may raise several questions regarding the generalizability of our findings. Additionally, we did not use any rating scale to specifically rate severity of BPD. Therefore, future FEP studies also exploring this crucial topic are needed (especially regarding relevant clinical features like self-harm and fear of abandonment that could be affected by transient psychotic breaks).

Second, we specifically examined FEP individuals in a “real-world” treatment setting primarily aimed at providing specialized EIP interventions within community mental healthcare services. Therefore, our findings should be compared to similar clinical populations.

Another limitation was related to the diagnostic assessment procedure. In this investigation, the DSM-IV-TR diagnoses were reformulated after a 2-year follow-up period, and only participants with BPD as final diagnosis were included in the FEP/BPD subgroup. Our results must be compared to similar FEP/BPD populations. Comparison difficulties could also arise using other assessment strategies to categorize FEP patients with BPD (such as the BPD screening instrument, not using a clinical interview method to differentiate the categorical disorder from its subthreshold features) [2]. Moreover, it should be considered that patients with schizotypal personality disorder were included in the FEP/SSD subgroup, according to a more stringent conceptualization of the schizophrenia spectrum [79].

Furthermore, although the DUP usually is 1 to 2 years before access to care in the EIP research paradigm [27], this often is not a criterion for exclusion from integrating an early intervention program in real-world clinical settings. Indeed, the delay in access to care should not penalize FEP patients, also raising an ethical question. Therefore, our findings are not generalizable outside similar clinical samples.

Additionally, another weakness was related to the criterion for excluding exposure to AP medication regardless of the dose. Indeed, when studying a population with BPD individuals, BPD condition may have been expressed before the FEP and have justified a prescription of AP drug even without psychotic symptoms. This treatment is often used in this specific population even if it does not correspond to the recommendations of good practice. However, as AP prescription was not often clearly justified and understandable from FEP patient’s clinical interviews or clinical charts, we preferred to strictly refer to the original definition of FEP threshold provided in the CAARMS (i.e., “essentially that at which an antipsychotic treatment would probably be started in the common clinical practice”). This certainly excluded some BPD patients using AP medications outside psychotic symptoms but avoided to include BPD individuals with a history of past psychotic episode not clearly detected from patients’ interviews or consulting their clinical charts.

Moreover, the fact of not having looked in this research at the proportion of participants who rated positive for the criteria of both BDP and SDD could be another limit to add. In this respect, several investigations that compared clinical populations of BPD and SDD patients highlighted that a significant part of their samples (often ranging between 15 and 20%) had the disorders occurring [68–71]. Therefore, future studies also exploring this occurrence in FEP individuals are needed.

Finally, the current study was designed within a specialized EIP program not specifically focused on BPD in FEP. Specifically, BPD psychopathology was not longitudinally assessed. Therefore, future perspective studies exploring BPD symptoms with more specific assessment instruments (such as the “BPD Checklist”) [80] are needed.

## Conclusions

BPD as categorical disorder may involve a not negligible part of FEP patients enrolled within specialized EIP services. These FEP patients with BPD seem to represent a FEP subgroup with specific clinical characteristics and challenges (e.g., high rates of substance abuse and drop-out, low levels of negative symptoms, higher occupational functioning at entry), differentiating it from SSD. Traditional EIP interventions appear to be less effective in these FEP/BPD subjects compared to FEP/SSD ones.

The results of this investigation have some crucial benefits for clinical practice. First, mental health professionals should pay attention to detect BPD already at the recruitment of young FEP patients in specialized EIP services. Indeed, the early identification of a BPD diagnosis in FEP individuals would allow detecting a group of patients at higher risk of service disengagement and potentially less responsive to specialized EIP interventions. However, future longitudinal research establishing appropriate treatment guidelines for this complex patient group is needed.

## Supplementary Information

Below is the link to the electronic supplementary material.

Supplementary file1 (DOC 122 KB)

## Data Availability

The data that support the findings of this investigation are available on reasonable request from the corresponding author. The data are not directly available due to privacy/ethical restrictions.
