# Supplementary material for: Borderline personality disorder vs. schizophrenia spectrum disorders in young people recruited within an “Early Intervention in Psychosis” service: clinical and outcome comparisons
Source: Eur Arch Psychiatry Clin Neurosci. 2024 Mar 12;275(3):893–905. doi: 10.1007/s00406-024-01772-5 (PMC11946946; doi:10.1007/s00406-024-01772-5)
Supplement: Supplementary file 1 — Supplementary file1 (DOC 122 KB) [file 406_2024_1772_MOESM1_ESM.doc]

Table S1 – Longitudinal data: Strobe flow chart.

49 BPD

356 FEP participants

307 SSD

45 Drop-out

↓

18 T1

27 T2

246 2-year PrEP termination

31 Conclusion

Other DMH

↓

20 T1

11 T2

34 Conclusion

Clinical improvement (discharge)

↓

7 T1

27 T2

49 BPD participants

65 Early PrEP termination

↓

27 T1

38 T2

11 Drop-out

↓

3 T1

8 T2

15 Early PrEP termination

↓

9 T1

6 T2

23 2-year PrEP termination

8 Conclusion

Clinical improvement (discharge)

↓

5 T1

3 T2

7 Conclusion

(outside the Pr-EP catchment area)

↓

4 T1

3 T2

Legend. FEP = First Episode Psychosis, BPD = Borderline Personality Disorder, SSD = Schizophrenia Spectrum Disorder; Pr-EP = Parma Early Psychosis program, T1 = 1-year assessment time, T2 = 2-year assessment time.

Table S2 – Flowchart for sampling method.

356 FEP participants

Baseline (T0)

Baseline diagnosis

49 BPD

2-year assessment time (T2)

Final diagnosis

307 SSD

*Drop-out*

As for participants who did not complete the follow-up period, final diagnoses were defined together with clinicians treating and managing FEP patients.

Legend. FEP = First Episode Psychosis, BPD = Borderline Personality Disorder, SSD = Schizophrenia Spectrum Disorder; T0 = baseline assessment time, T2 = 2-year assessment time.

Table S3 – DSM-IV-TR diagnosis at baseline in the two FEP subgroups (n = 356).

| Variable | BPD  (n = 49) | SSD  (n = 307) |
| --- | --- | --- |
| Schizophreniform disorder  Drug-induced psychotic disorder  Psychotic disorder not otherwise specified  Brief psychotic disorder  Affective psychosis  Schizophrenia  Schizo-affective disorder  Delusional disorder  Schizotypal personality disorder | 13 (26.5%)  13 (26.5%)  11 (22.4%)  10 (20.4%)  2 (4.0%)  -  -  -  - | 47 (15.3%)  2 (0.6%)  38 (12.4%)  25 (8.1%)  6 (2.0%)  155 (50.5%)  11 (3.6%)  15 (4.9%)  8 (2.6%) |

Note. DSM-IV-TR = Diagnostic and Statistical Manual of mental disorders, IV Edition, Text Revision; FEP = First Episode Psychosis; BPD = Borderline Personality Disorder; SSD = Schizophrenia Spectrum Disorders. Frequencies and percentages are reported.

Table S4 – Kaplan-Meier survival analysis results: comparison on 2-year drop-out rate between the two FEP subgroups (n = 356).

| Time intervals  (in months) | | BPD (n = 49) | | | | | | | | | | | SSD (n = 307) | | | | |
| --- | --- | --- | --- | --- | --- | --- | --- | --- | --- | --- | --- | --- | --- | --- | --- | --- | --- |
|  |  | Cumulative Proportion  Surviving at the Time | | | | | | Cumulative events  (n) | | | Censored  (n) | | Cumulative Proportion  Surviving at the Time | | | Cumulative events  (n) | Censored  (n) |
|  |  | Estimate | | | SE | | |  |  |  |  |  | Estimate | | SE |  |  |
| 0-6  6-12  12-18  18-24 | | 0.980  0.929  0.725  0.695 | | | 0.020  0.040  0.075  0.078 | | | 1  3  10  11 | | | 9  17  25  49 | | 0.967  0.950  0.885  0.877 | | 0.010  0.013  0.019  0.020 | 10  15  32  34 | 24  55  81  307 |
| FEP  subgroup | Mean (in months) | | | | | | | | | | | | |  | | | |
|  | Estimate | | | SE | | | 95% CI | | | | | | |  | | | |
|  |  |  |  |  |  |  | Lower bound | | | Upper bound | | | |  | | | |
| BPD  SSD  Overall | 20.946  23.657  22.341 | | | 0.880  0.262  0.252 | | | 19.221  22.006  21.847 | | | 22.671  23.034  22.834 | | | |  | | | |
|  | | | Χ^2^ | | | df | | | p | | |  | | | | | |
| Log Rank (Mantel-Cox) | | | 7.566 | | | 1 | | | **0.006** | | |  | | | | | |

Legend. BPD = Borderline Personality Disorder; SSD = Schizophrenia Spectrum Disorder; SE = Standard Error; 95% CI = 95% Confidence Intervals; Log Rank = Logarithm Rank Test; = Chi-Square test; df = degrees of freedom; p = statistical value. Significant statistical p values are in bold.

Table S5 – Kaplan-Meier survival analysis results: comparison on 2-year new hospitalization rate between the two FEP subgroups (n = 356).

| Time intervals  (in months) | | BPD (n = 49) | | | | | | | | | | | SSD (n = 307) | | | | |
| --- | --- | --- | --- | --- | --- | --- | --- | --- | --- | --- | --- | --- | --- | --- | --- | --- | --- |
|  |  | Cumulative Proportion  Surviving at the Time | | | | | | Cumulative events  (n) | | | Censored  (n) | | Cumulative Proportion  Surviving at the Time | | | Cumulative events  (n) | Censored  (n) |
|  |  | Estimate | | | SE | | |  |  |  |  |  | Estimate | | SE |  |  |
| 0-12  12-24 | | 0.846  0.846 | | | 0.058  0.058 | | | 6  6 | | | 12  49 | | 0.766  0.708 | | 0.025  0.028 | 65  78 | 87  307 |
| FEP  subgroup | Mean (in months) | | | | | | | | | | | | |  | | | |
|  | Estimate | | | SE | | | 95% CI | | | | | | |  | | | |
|  |  |  |  |  |  |  | Lower bound | | | Upper bound | | | |  | | | |
| SSD  BPD  Overall | 21.194  22.154  21.312 | | | 0.307  0.693  0.283 | | | 20.593  20.795  20.758 | | | 21.795  23.513  21.866 | | | |  | | | |
|  | | | Χ^2^ | | | df | | | p | | |  | | | | | |
| Log Rank (Mantel-Cox) | | | 2.368 | | | 1 | | | 0.124 | | |  | | | | | |

Legend. BPD = Borderline Personality Disorder; SSD = Schizophrenia Spectrum Disorder; SE = Standard Error; 95% CI = 95% Confidence Intervals; Log Rank = Logarithm Rank Test; = Chi-Square test; df = degrees of freedom; p = statistical value. Significant statistical p values are in bold.

Table S6 – Kaplan-Meier survival analysis results: comparison on 2-year new attempted suicide rate between the FEP two subgroups (n = 356).

| Time intervals  (in months) | | BPD (n = 49) | | | | | | | | | | | SSD (n = 307) | | | | |
| --- | --- | --- | --- | --- | --- | --- | --- | --- | --- | --- | --- | --- | --- | --- | --- | --- | --- |
|  |  | Cumulative Proportion  Surviving at the Time | | | | | | Cumulative events  (n) | | | Censored  (n) | | Cumulative Proportion  Surviving at the Time | | | Cumulative events  (n) | Censored  (n) |
|  |  | Estimate | | | SE | | |  |  |  |  |  | Estimate | | SE |  |  |
| 0-12  12-24 | | 0.949  0.862 | | | 0.035  0.066 | | | 2  4 | | | 9  49 | | 0.953  0.949 | | 0.013  0.013 | 13  14 | 36  307 |
| FEP  subgroup | Mean (in months) | | | | | | | | | | | | |  | | | |
|  | Estimate | | | SE | | | 95% CI | | | | | | |  | | | |
|  |  |  |  |  |  |  | Lower bound | | | Upper bound | | | |  | | | |
| SSD  BPD  Overall | 23.439  23.385  23.432 | | | 0.158  0.489  0.147 | | | 23.130  22.425  23.144 | | | 23.748  24.344  23.721 | | | |  | | | |
|  | | | Χ^2^ | | | df | | | p | | |  | | | | | |
| Log Rank (Mantel-Cox) | | | 1.972 | | | 1 | | | 0.160 | | |  | | | | | |

Legend. BPD = Borderline Personality Disorder; SSD = Schizophrenia Spectrum Disorder; SE = Standard Error; 95% CI = 95% Confidence Intervals; Log Rank = Logarithm Rank Test; = Chi-Square test; df = degrees of freedom; p = statistical value. Significant statistical p values are in bold.

Table S7 – Kaplan-Meier survival analysis results: comparison on 2-year new self-harm behavior rate between the two subgroups (n = 356).

| Time intervals  (in months) | | BPD (n = 49) | | | | | | | | | | | SSD (n = 307) | | | | |
| --- | --- | --- | --- | --- | --- | --- | --- | --- | --- | --- | --- | --- | --- | --- | --- | --- | --- |
|  |  | Cumulative Proportion  Surviving at the Time | | | | | | Cumulative events  (n) | | | Censored  (n) | | Cumulative Proportion  Surviving at the Time | | | Cumulative events  (n) | Censored  (n) |
|  |  | Estimate | | | SE | | |  |  |  |  |  | Estimate | | SE |  |  |
| 0-12  12-24 | | 0.821  0.772 | | | 0.061  0.074 | | | 7  8 | | | 10  49 | | 0.849  0.818 | | 0.021  0.024 | 42  49 | 29  307 |
| FEP  subgroup | Mean (in months) | | | | | | | | | | | | |  | | | |
|  | Estimate | | | SE | | | 95% CI | | | | | | |  | | | |
|  |  |  |  |  |  |  | Lower bound | | | Upper bound | | | |  | | | |
| SSD  BPD  Overall | 22.187  21.846  22.145 | | | 0.260  0.788  0.246 | | | 21.677  20.301  21.663 | | | 22.697  23.391  22.627 | | | |  | | | |
|  | | | Χ^2^ | | | df | | | p | | |  | | | | | |
| Log Rank (Mantel-Cox) | | | 0.350 | | | 1 | | | 0.554 | | |  | | | | | |

Legend. BPD = Borderline Personality Disorder; SSD = Schizophrenia Spectrum Disorder; SE = Standard Error; 95% CI = 95% Confidence Intervals; Log Rank = Logarithm Rank Test; = Chi-Square test; df = degrees of freedom; p = statistical value. Significant statistical p values are in bold.
